# A pan-sarcoma landscape of telomeric content shows that alterations in *RAD51B* and *GID4* are associated with higher telomeric content

**DOI:** 10.1038/s41525-023-00369-6

**Published:** 2023-09-14

**Authors:** Radwa Sharaf, Dexter X. Jin, John Grady, Christine Napier, Ericka Ebot, Garrett M. Frampton, Lee A. Albacker, David M. Thomas, Meagan Montesion

**Affiliations:** 1 Foundation Medicine Inc., Cambridge, MA USA; 2 Omico Australian Genomic Cancer Medicine, Sydney, Australia; 3 Garvan Institute of Medical Research, Sydney, Australia; 4 St Vincent’s Clinical School, University of New South Wales, Sydney, Australia

**Keywords:** Medical genomics, Sarcoma

## Abstract

Tumor cells need to activate a telomere maintenance mechanism, enabling limitless replication. The bulk of evidence supports that sarcomas predominantly use alternative lengthening of telomeres (ALT) mechanism, commonly associated with alterations in *ATRX* and *DAXX*. In our dataset, only 12.3% of sarcomas harbored alterations in these genes. Thus, we checked for the presence of other genomic determinants of high telomeric content in sarcomas. Our dataset consisted of 13555 sarcoma samples, sequenced as a part of routine clinical care on the FoundationOne®Heme platform. We observed a median telomeric content of 622.3 telomeric reads per GC-matched million reads (TRPM) across all samples. In agreement with previous studies, telomeric content was significantly higher in *ATRX* altered and *POT1* altered sarcomas. We further observed that sarcomas with alterations in *RAD51B* or *GID4* were enriched in samples with high telomeric content, specifically within uterus leiomyosarcoma for *RAD51B* and soft tissue sarcoma (not otherwise specified, nos) for *GID4*, Furthermore, *RAD51B* and *POT1* alterations were mutually exclusive with *ATRX* and *DAXX* alterations, suggestive of functional redundancy. Our results propose a role played by *RAD51B* and *GID4* in telomere elongation in sarcomas and open research opportunities for agents aimed at targeting this critical pathway in tumorigenesis.

## Introduction

Sarcomas represent a rare and heterogeneous group of tumors with a mesenchymal origin, exhibiting different biological behavior and subsequent varying clinical manifestations. Due to their rarity, they have been relatively understudied. To allow for unchecked cellular proliferation, tumors must overcome the problem of telomere shortening^1^. Telomeres are DNA-protein complexes that consist of 5–15 kb of repetitive hexamers forming protective caps at the ends of linear chromosomes and shorten by an average of 50–150 base pairs with every cell cycle^2–5^. If telomeres reach a critical limit in length, cells go into replicative senescence and can no longer divide^6,7^.

Currently, two mechanisms for telomere maintenance have been described. These include telomerase-dependent elongation, mediated by *TERT* activation^8–11^, and alternative lengthening of telomeres (ALT), linked to the loss of *ATRX* or *DAXX*^12–14^. Conflicting results have been published about telomerase activity in sarcomas, exemplified by the wide range of reported soft tissue sarcomas displaying telomerase activity, ranging from 7% up to 81%^15–18^. Previous studies have reported a *TERT* promoter mutation prevalence rate of 6–11% across sarcomas^19–21^. The bulk of evidence supports that 20–60% of sarcomas activate the ALT pathway for telomere elongation^22–26^. However, in our dataset, only 12.3% of sarcomas harbored *ATRX*/*DAXX* alterations (11.4% for *ATRX* and 0.9% for *DAXX*), leaving the remaining cases unexplained.

Here we sought to investigate the genomic determinants of high telomeric content beyond the canonical *ATRX*/*DAXX* alterations within our sarcoma dataset. We characterized the telomeric landscape of 13555 sarcoma samples across 38 disease types sequenced on the FoundationOne®Heme platform. We also validated our findings using samples sequenced on a different platform, FoundationOne®CDx, as well as samples from an independent academic cohort. To assess potential clinical relevance, we compared overall survival from date of metastatic diagnosis of sarcoma patients whose tumors harbored genomic alterations associated with high telomeric content with patients whose tumors lacked these alterations.

## Results

### Telomeric content across FMI’s sarcoma cohort

**Fig. 1:**
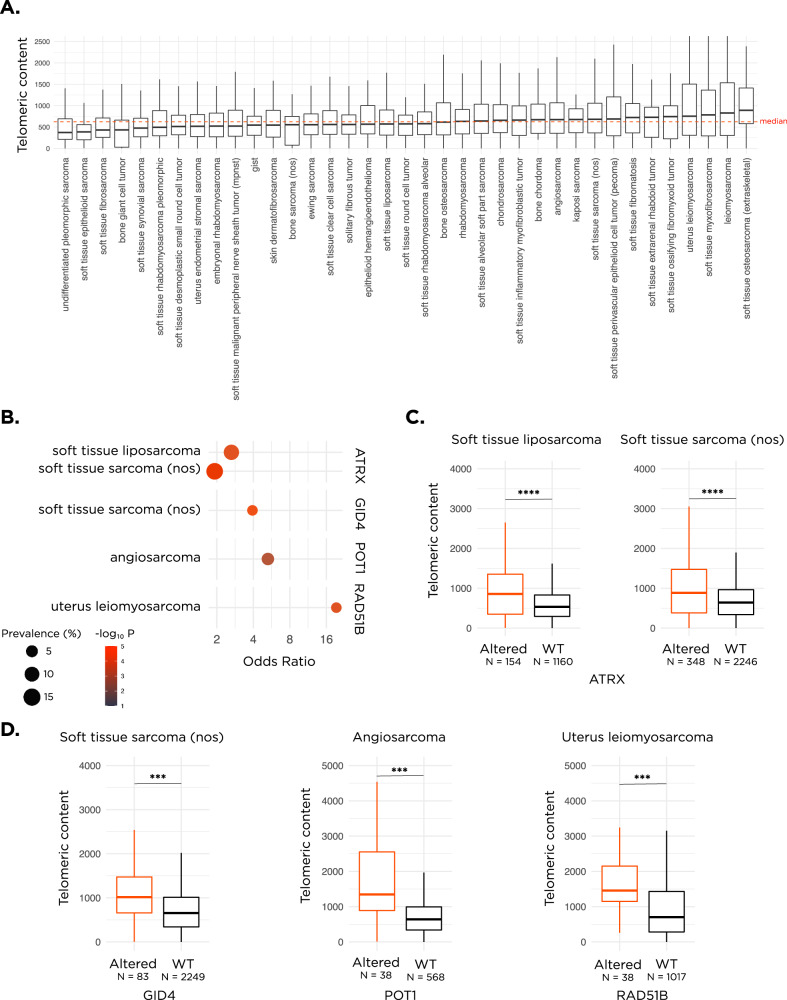
Overview of telomeric content landscape in sarcomas and screen results. **A** A boxplot showing the landscape of telomeric content across sarcoma disease groups. The red dotted line depicts the median telomeric content across all samples. **B** Results from the screen displaying the 4 genetic associations identified within each disease ontology. The *p*-value is denoted by the color and alteration prevalence is denoted by the size of each circle. **C** Boxplots depicting the difference in telomeric content between *ATRX* altered and *ATRX* WT samples within soft tissue liposarcoma and soft tissue sarcoma nos. **D** Boxplots depicting the difference in telomeric content between *GID4* altered vs *GID4* WT samples within soft tissue sarcoma nos, *POT1* altered vs *POT1* WT within angiosarcoma, and *RAD51B* altered vs *RAD51B* WT within uterus leiomyosarcoma. The counts of samples within each group are indicated under the *x*-axis. ****p < 0.0001 and ***p < 0.001. nos, not otherwise specified. In all boxplots, the box extends from the first to the third quartile with a line in the middle that represents the median.

We measured the telomeric content for 13555 sarcoma samples, representing 38 unique sarcoma types, using TelomereHunter. Our results showed that telomeric content varied by sarcoma disease type, with a median telomeric content of 622.3 TRPM (telomeric reads per GC content-matched million reads) across all sarcoma types and a range of 519.4 (Fig. 1A and Table S1). The highest median telomeric content was observed in soft tissue osteosarcoma (extraskeletal) at 892.4 TRPM and the lowest in undifferentiated pleomorphic sarcoma at 373.0 TRPM. Based on previously-published criteria^27,28^, we categorized sarcoma diseases broadly as either translocation-associated (*N* = 2447, 18.1%) or genomically complex/other which display complex karyotypes (*N* = 11108, 82.0%, Table S1). We hypothesized that the median telomeric content of the genomically complex sarcomas will be greater than the translocation-associated group, given the extra telomeres stemming from chromosome gain events. Our analysis showed that both the median telomeric content, as well as the median age, of the genomically complex/other group was significantly higher than the translocation-associated group (650 vs 542 TRPM, *p* < 0.0001 for telomeric content and 56 vs 36 years, *p* < 0.0001 for age, Fig. S1A, B). This could explain our observation that sarcoma samples from older patients had a higher median telomeric content (Fig. S1C).

### Impact of genetic alterations on telomeric content

To assess which genetic alterations were associated with high telomeric content, we compared the frequency of alterations observed in samples that fall in the top quartile of telomeric content to the frequency seen in the bottom quartile within each tumor type. In agreement with previous studies, telomeric content was significantly higher in *ATRX* altered samples. Specifically, alterations in *ATRX* were significantly enriched in the top quartile of soft tissue liposarcoma (Bonferroni-corrected *p* < 0.01, odds ratio (OR) = 2.7 [1.7–4.2]) and soft tissue sarcoma not otherwise specified (nos, Bonferroni-corrected *p* < 0.01, OR = 2.0 [1.4–2.6], Fig. 1B and Table S2). Median telomeric content of *ATRX* altered samples was significantly higher than WT samples (877 vs 534 TRPM for soft tissue liposarcoma and 889 vs 647 TRPM for soft tissue sarcoma nos, Fig. 1C). Intriguingly, *DAXX* did not show up in our screen, so we assessed the impact of its alterations on the telomeric content of three disease types with at least 20 *DAXX* altered samples. The median telomeric content was higher in two groups but did not reach statistical significance after Bonferroni’s correction (1041 vs 730 TRPM for uterus leiomyosarcoma and 1132 vs 671 TRPM for soft tissue sarcoma nos, *p* > 0.05 for all comparisons, Fig. S2).

We further identified that alterations in 3 genes, *GID4*, *POT1*, and *RAD51B*, were enriched in sarcomas with high telomeric content, specifically within soft tissue sarcoma nos for *GID4* (Bonferroni-corrected *p* < 0.01, OR = 3.9 [2.0–7.7]), angiosarcoma samples for *POT1* (Bonferroni-corrected *p* < 0.05, OR = 5.3 [2.0–14.4]), and uterus leiomyosarcoma for *RAD51B* (Bonferroni-corrected *p* < 0.01, OR = 20.5 [2.6–146.0], Fig. 1B and Table S2). Interestingly, the odds ratio for the enrichment of alterations within these three newly-identified genes in high telomeric content samples exceeded the odds ratio observed for *ATRX*, a canonical telomere maintenance mechanism (TMM) gene, alterations in which are known to be associated with ALT. The impact of alterations in *RAD51B*, *GID4*, and *POT1* on telomeric content was proportional to tumor purity, but in samples WT for known TMM genes, telomeric content was not impacted by tumor purity (Fig. S3). Median telomeric content of *GID4* altered soft tissue sarcoma nos samples was significantly higher than *GID4* WT samples (1016 vs 662 TRPM, *p* < 0.001), *POT1* altered angiosarcoma was significantly higher than *POT1* WT (1485 vs 651 TRPM, *p* < 0.001), and *RAD51B* altered uterus leiomyosarcoma was significantly higher than *RAD51B* WT (1529 vs 710 TRPM, *p* < 0.001, Fig. 1D). Overall, the alteration prevalence for these genes was low, where *GID4* was altered in 3.6% (83/2332) of soft tissue sarcoma nos, *POT1* in 6.3% (38/606) of angiosarcoma, and *RAD51B* in 3.6% (38/1055) of uterus leiomyosarcoma samples (Table S3).

### Types of genetic alterations in *RAD51B*, *GID4*, and *POT1*

**Fig. 2:**
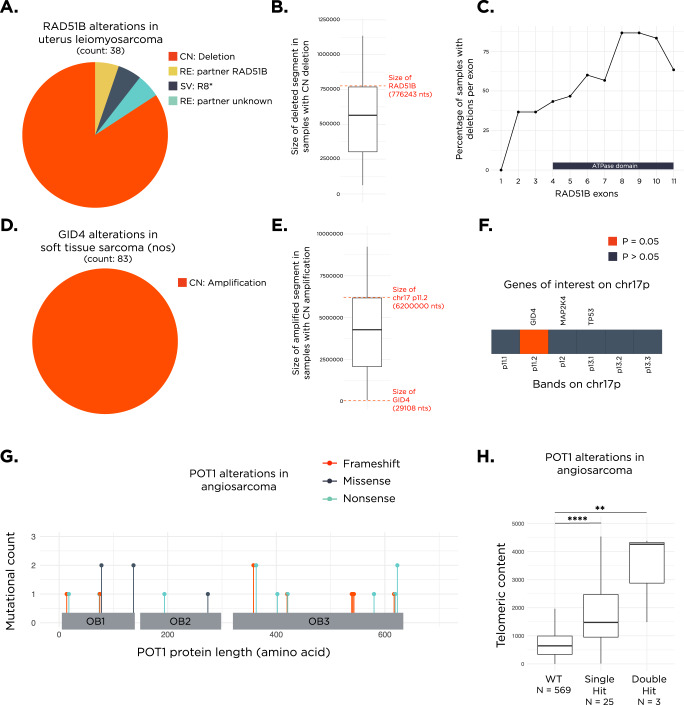
Alteration types observed for RAD51B, GID4, and POT1. **A** Pie chart displaying the proportion of various *RAD51B* alterations observed across uterus leiomyosarcoma samples. **B** Within samples with a *RAD51B* copy-number deletion, the size of the deleted segment is displayed on the boxplot and a red dotted line denotes the size of the *RAD51B* gene in nucleotides. **C** A line plot displaying the proportion of samples with a *RAD51B* copy-number deletion harboring deletions within each exon of RAD51B. The position of the ATPase domain is indicated at the bottom. **D** Pie chart showing that all of the *GID4* alterations observed across soft tissue sarcoma nos samples are copy number amplifications. **E** Within samples with a *GID4* copy-number amplification, the size of the amplified segment is displayed on the boxplot and two red dotted lines indicate the size of the *GID4* gene and the size of the chr17p11.2 cytoband, respectively. **F** Analysis of enrichment for high telomeric content in samples with amplifications across chromosome bands in chr17p. Select genes of interest within certain cytobands are shown on the top. Orange indicates *p* = 0.05 and slate blue indicates *p* > 0.05. **G** Lollipop plot displaying the count of mutations observed across the Oligonucleotide/Oligosaccharide Binding (OB) fold domains of *POT1*. **H** Boxplot showing the distribution of telomeric content values across angiosarcoma samples with a *POT1* single hit mutation, double hit mutation or WT. CN copy number alteration, RE rearrangement, SV short variant alteration, nos, not otherwise specified, nts nucleotides, TPM transcripts per million. ****p < 0.0001 and **p < 0.01. In all boxplots, the box extends from the first to the third quartile with a line in the middle that represents the median.

Within uterus leiomyosarcomas, 84.2% of the identified *RAD51B* alterations were copy number deletions (Fig. 2A). Median size of the deleted segment was 565171 nucleotides, which is smaller than the size of *RAD51B* at 776243 nucleotides (Fig. 2B). Thus, we investigated which region of *RAD51B* was most frequently deleted and observed that in the majority of samples, the deletion spanned exons 4–11 corresponding to the ATPase domain (Fig. 2C)^29^. We also observed that *RAD51B* alterations were commonly co-occurring with alterations in *FAF1* (26.5%), *CDKN2C* (23.5%), and *CIC* (14.7%, Fig. S4).

Within soft tissue sarcoma nos, all the *GID4* alterations were copy number amplifications with a median copy number of 7 (range 5–45 copies, Fig. 2D). Median size of the amplified segment was 4320468 nucleotides, which is smaller than the size of chr17p11.2 (6.2 Mb) where *GID4* lies, but larger than the size of *GID4* itself at 29108 nucleotides (Fig. 2E). We also assessed whether the amplification of any of the cytobands on the p-arm of chr17 was associated with high telomeric content in soft tissue sarcoma nos. Our analysis showed that samples with amplifications in p11.2 on chr17 were enriched in samples with high telomeric content (*p* = 0.05, Fig. 2F). This association was not observed in any of the other cytobands on chr17p. We also observed that *GID4* altered samples were associated with alterations in *TP53* (83.3%) and *RB1* (47.8%, Fig. S4).

Within angiosarcoma, all the *POT1* alterations were short variant alterations, occurring throughout its length, where 70% of the mutations were observed only once and none were observed more than twice (Fig. 2G). For samples where zygosity could be assessed, the majority of samples (89%, 25/28) harbored a single-hit *POT1* alteration and in 11% (3/28) of samples, double-hit mutations were observed. One particular sample with a double-hit alteration had one mutation predicted to be somatic and another predicted to be of germline origin, while the remaining two samples with double-hit alterations had predicted homozygous somatic mutations. Median telomeric content was higher in the double-hit group vs single-hit but did not reach statistical significance (4266 vs 1570 TRPM, *p* > 0.05). However, both groups had significantly higher telomeric content compared to WT (median WT: 652 TRPM, *p* < 0.01 for double-hit, *p* < 0.0001 for single-hit, Fig. 2H). We also observed that *POT1* altered samples were enriched in samples with a high tumor mutational burden (TMB) and harboring a UV mutational signature (Fig. S4), similar to findings from an angiosarcoma landscape study which showed *POT1* alterations to be enriched in TMB high head and neck angiosarcomas^30^.

### Mutual exclusivity of genetic alterations and impact of co-occurrence

**Fig. 3:**
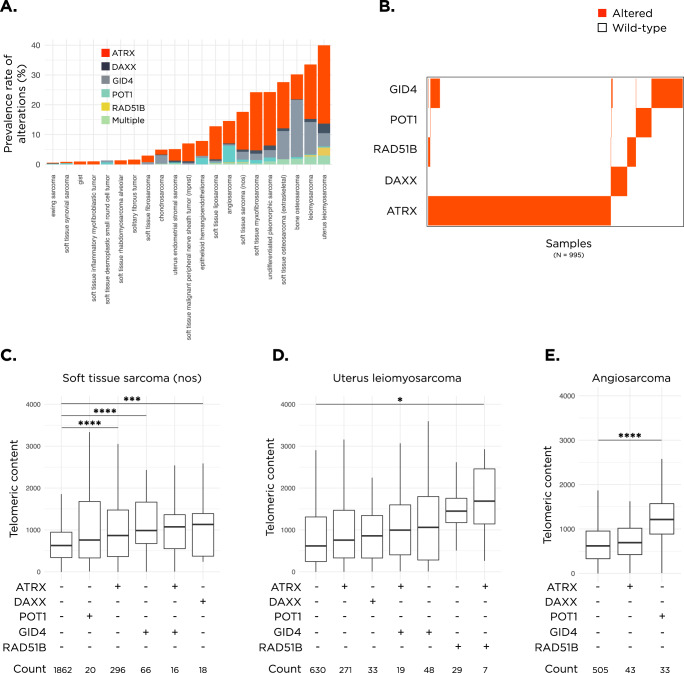
Mutual exclusivity of RAD51B, POT1, ATRX, and DAXX. **A** Barplot indicating the prevalence rate of alterations within *ATRX*, *DAXX*, *GID4*, *POT1*, and *RAD51B* within each disease. Analysis restricted to diseases with at least 100 total samples. **B** Tile plot showing the distribution of alterations in *ATRX*, *DAXX*, *RAD51B*, *POT1*, and *GID4* within sarcoma samples in the FMI dataset. Plot depicts 995 sarcoma samples, which harbor at least one alteration in these genes. Altered samples are shown in orange and non-altered samples are shown as white. The impact of single and/or multiple alterations within these genes on telomeric content is shown for soft tissue sarcoma nos in **C**, uterus leiomyosarcoma samples in **D**, and angiosarcomas in **E**. Within each group, the symbol (+) means altered and the symbol (-) means non-altered. Groups are plotted if they contain >5 samples. Only comparisons against WT are shown. ****p < 0.001, ***p < 0.001, and *p < 0.05. In all boxplots, the box extends from the first to the third quartile with a line in the middle that represents the median.

Prevalence of *RAD51B*, *GID4*, and *POT1* alterations varied widely across sarcoma subtypes and tended to mostly occur in diseases with high prevalence rates of alterations in the other telomere maintenance genes (Fig. 3A). Thus, we checked for mutual exclusivity and observed that alterations within *RAD51B* and *POT1* were predominantly mutually exclusive with alterations in *ATRX* and *DAXX* (*p* = 8E-6, Fig. 3B). This is suggestive of a redundancy between alterations in these genes, likely a result of affecting similar pathways. *GID4*, on the other hand, was co-altered with *ATRX* in 23% of *GID4* altered samples (Fig. 3B). Consequently, we queried the impact of a *GID4* alteration on top of an existing *ATRX* alteration on telomeric content. In soft tissue sarcoma nos, median telomeric content of the double-altered samples was higher than either single *GID4* altered or single *ATRX* altered samples, although neither reached statistical significance (1072 vs 1053 vs 874 TRPM, respectively, *p* > 0.05 for all comparisons, Fig. 3C). Similarly, in *ATRX* altered uterus leiomyosarcoma, *RAD51B* alterations exerted an additive effect on telomeric elongation (2226 TRPM in double-altered vs 1449 in *RAD51B* altered only and 812 in *ATRX* altered only, Fig. 3D). Across three different disease ontologies, when samples were ranked based on median telomeric content, alterations in the newly identified genes from our screen (*RAD51B*, *GID4*, and *POT1*) were seen in the groups with the highest or second highest telomeric content, or both (Fig. 3C–E).

### Impact of alterations on TERRA levels

**Fig. 4:**
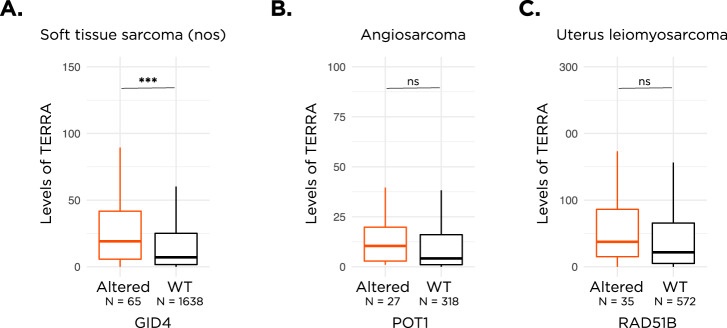
Levels of TERRA expression. Boxplots depicting differences in levels of TERRA expression in *GID4* altered vs *GID4* WT soft tissue sarcoma nos samples (**A**), *POT1* altered vs *POT1* WT angiosarcoma samples (**B**), and *RAD51B* altered vs *RAD51B* WT uterus leiomyosarcoma samples (**C**). ns not significant. ***p< 0.001. In all boxplots, the box extends from the first to the third quartile with a line in the middle that represents the median.

ALT has been linked to elevated levels of long noncoding telomeric repeat-containing RNA (TERRA), transcribed off telomeric repeats^31–34^. Thus, we measured TERRA levels using TelomereHunter for select samples with available RNA sequencing data and compared between TERRA levels in samples with alterations in *RAD51B*, *GID4*, or *POT1* vs WT. We observed that the median level of TERRA expression was significantly higher in *GID4* altered soft tissue sarcoma nos vs WT (Fig. 4A, 22.7 vs 9.1, *p* < 0.001). We also observed a trend towards higher median TERRA levels in *POT1* altered angiosarcoma vs WT (Fig. 4B, 12.5 vs 5.8, *p* > 0.05) and a trend towards higher levels in *RAD51B* altered uterus leiomyosarcoma vs WT (Fig. 4C, 46.7 vs 30.1, *p* > 0.05).

### Comparison of results to two independent datasets

**Fig. 5:**
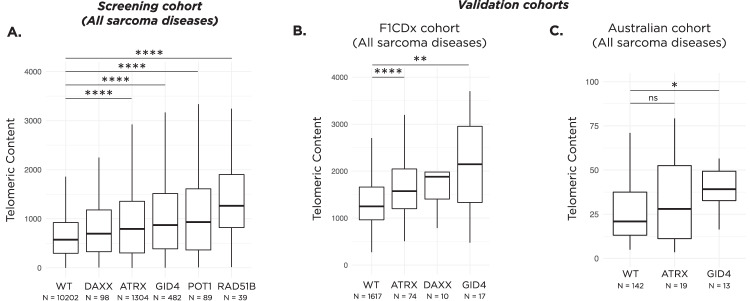
Impact of alterations on telomeres pan-sarcoma. **A** Boxplot displaying the distribution of telomeric content values of samples in our screening cohort across all sarcoma diseases. ****p < 0.0001. Analysis was restricted to samples that are altered in only one of the telomere-maintenance mechanism genes. **B** Boxplot of the telomeric content values in the F1CDx cohort, specifically for WT, *ATRX*, *DAXX*, and *GID4* altered soft tissue tumors. Analysis restricted to groups with at least 10 samples. **p < 0.01 and ****p < 0.0001. **C** Boxplot of the telomeric content values in the Australian cohort, specifically for WT, *ATRX*, and *GID4* altered soft tissue tumors. Analysis restricted to groups with at least 10 samples. *p < 0.05; ns not significant. In all boxplots, the box extends from the first to the third quartile with a line in the middle that represents the median.

Across all sarcoma samples within our screening cohort, samples with alterations in either *GID4*, *RAD51B*, *POT1*, or *ATRX* had significantly higher telomeric content than WT samples (Fig. 5A). All sarcoma samples in our screening cohort were tested using the FoundationeOne®Heme platform. A minority of the sarcoma samples received at our institution were run on a different testing platform, FoundationOne®CDx (F1CDx) and were not included in the screen. We thus used these 1739 F1CDx sarcoma samples for validation. We grouped samples based on the alteration status of their genes and compared between their telomeric content (Fig. 5B). Only groups with at least 10 samples are shown. Alterations in *ATRX* and *GID4* were associated with a significantly higher telomeric content compared to WT samples (median telomeric content 1263 TRPM in WT, 2146 TRPM in *GID4* altered, and 1646 TRPM in *ATRX* altered). In addition, we conducted a disease-specific analysis for the F1CDx cohort based on the sarcoma subtypes identified in our screen and observed that the results trended in the same direction (Fig. S5).

We also investigated the impact of genetic alterations on the telomeric content in an independent Australian dataset consisting of 174 sarcoma samples profiled on the TSO500 assay. *GID4* altered samples had significantly higher median telomeric content compared to WT (40.9 vs 22.6 TRPM, *p* < 0.05, Fig. 5C), while the median telomeric content of *ATRX* altered samples trended towards being higher (29.1 vs 22.6 TRPM, *p* > 0.05, Fig. 5C). Only groups with at least 10 samples were included in this analysis. Of note, panels from both independent validation datasets contained baiting for *TERC* and *TERT*. None of the samples altered in *RAD51B*, *GID4*, *ATRX*, or *DAXX* harbored alterations in *TERC* or *TERT*.

### Impact of GID4 alterations on overall survival in sarcoma

Next, we checked whether the presence of *GID4* alterations impacted overall survival in sarcoma patients with metastatic disease. To this purpose, we utilized two clinical cohorts. The first is the CGDB cohort, which consisted of 449 sarcoma patients with metastatic disease, 213 of which were soft tissue sarcoma patients. The second is the Australian cohort, which consisted of 235 sarcoma patients with metastatic disease, 211 of which were soft tissue sarcoma patients. Patient demographics data is summarized in Table S4. No significant differences were observed in the mOS between *GID4* altered and *GID4* WT advanced sarcoma patients in either cohort (CGDB cohort: mOS of 5.6 months [3.0-NA] vs 9.8 months [7.4–12.2], *p* = 0.4; Australian cohort: mOS of NA months [19.7-NA] vs 41.3 months [30.9–59.9], *p* = 0.6, Fig. S6).

Simiarily, when the analysis was restricted to patients with soft tissue sarcoma tumors, no significant differences were observed in the mOS between *GID4* altered and *GID4* WT patients in either cohort (CGDB cohort: mOS of 3.5 months [1.6-NA] vs 8.1 months [5.6–11.4], *p* = 0.2; Australian cohort: mOS of NA months [35.8-NA] vs 41.3 months [34.1–59.9], *p* = 0.4, Fig. S6).

## Discussion

In this study, we investigated the genetic determinants of high telomeric content across 13555 sarcoma samples in the Foundation Medicine dataset and found that samples with high telomeric content were enriched in deletions in *RAD51B*, amplifications in *GID4* (17p11.2), and short variant mutations in *POT1* within select disease ontologies. This finding was largely in agreement with results from an additional dataset of sarcomas profiled on another Foundation Medicine platform, as well as in an independent academic dataset profiled on a different commercial platform.

Among sarcomas, multiple studies reported that a substantial proportion activates the ALT mechanism for telomere elongation^23,25^, which is associated with the loss of *ATRX* and *DAXX*^31,35^. However, it was also reported that *ATRX*/*DAXX* alterations were not present in all ALT-positive samples, with one study reporting that 45% of ALT-positive sarcoma samples were *ATRX* intact^23,36^. In our dataset, only 12.3% of sarcomas harbored *ATRX*/*DAXX* alterations. Given the necessity to elongate telomeres for tumorigenesis, we hypothesized that the remaining 87.7% of sarcoma samples need to have either acquired mutations in other genes leading to ALT or activated non-ALT-mediated mechanisms of telomere lengthening.

Our screen identified three genes: *RAD51B*, *POT1*, and *GID4*, alterations in which were associated with higher telomeric content. *RAD51B* is a DNA-damage repair pathway gene. It is a paralog of *RAD51*, sharing a low level of sequence identity with *RAD51* (∼25%), where most of the similarity is found within their ATPase domains^29,37^. RAD51’s activity and its role in homologous recombination repair appear to be strictly regulated by a number of cofactors including RAD51B^38,39^. Specifically, RAD51B is a component of the BCDX2 complex, that is responsible for the recruitment of RAD51 in the early stages of homologous recombination^40^. RAD51 has been identified in ALT-associated promyelocytic leukemia (PML) bodies (APBs)^41^. Recently, in vitro studies showed that RAD51 antagonizes the formation of C-circles^42^, which are extrachromosomal circular telomeric DNA considered a quantitative biomarker of the ALT mechanism^43,44^. However the depletion of RAD51 did not significantly reduce telomere DNA synthesis^42^.

In cell lines, *RAD51B* inactivation led to a comparatively mild phenotype, however the inactivation of other BCDX2 complex components resulted in a dramatic reduction in DNA double-strand break-induced homologous recombination^27,28^. A recent cryo-EM study observed that entirely different surfaces of RAD51B, RAD51C, and RAD51D are involved in intermolecular interactions within BCDX2^29^. To our knowledge, no studies have probed the impact of *RAD51B* deletion on telomere length. Our results show that its deletion is associated with higher telomeric content, suggesting that RAD51B may be a repressor of the BCDX2 complex. Further studies are needed to delineate the precise roles of RAD51 and RAD51B in telomere elongation.

POT1 is a component of the shelterin complex and binds to single-stranded telomeric DNA at the ends of linear chromosomes^45^. It was observed that heritable defects in *POT1* increase the risk of cancers, including sarcomas, and study participants with *POT1* mutations had significantly longer telomeres than age-matched controls^46–52^. In vitro cell line studies showed that POT1 modulates telomerase-mediated telomere elongation^53,54^. In human embryonic stem cells, known to express telomerase^55^, introducing mutations in *POT1* resulted in elongated telomeres^56^. The induction of POT1 ubiquitination and subsequent degradation induced cell death in ALT-positive cell lines, but was compatible with survival in telomerase-positive cells^57^.

No prior studies have suggested a link between GID4 and telomeres. GID4 is a subunit of the GID ubiquitin ligase, which plays a role in glucose sensing and energy homeostasis^58,59^. Others have reported that glucose levels in healthy individuals are negatively associated with telomere length^60^. Furthermore, patients with type 2 diabetes harbor shorter telomeres^61–64^ and their telomere length was associated with glycemic progression and diabetic complications, such as nephropathy^65–67^. In prospective studies where healthy non-diabetic participants were followed up for years, those with a lower telomere length at baseline were more likely to develop diabetes, with one study reporting a hazard ratio of 2.0 when comparing participants in the bottom vs top quartile of telomere length^68–70^. Consequently, it will be important to investigate the mechanisms mediating the interaction between telomere length and glucose metabolism. To our knowledge, no other genes on chr17p11.2 have been previously associated with telomere elongation either.

Overall, we observed a low alteration prevalence for the genes identified in our screen. *RAD51B* was altered in 3.6% (38/1055) of uterus leiomyosarcoma samples, lower than the previously reported *RAD51B* alteration prevalence of 7.4% (16/216)^71^ and 12.4% (13/105)^72^. Similarly, we observed a *POT1* alteration prevalence of 6.3% (38/606) in angiosarcoma samples, lower than previously reported at 15.7% (22/140) and 23.3% (20/86)^30,73^. It is important to note that despite the low alteration prevalence we observed for these genes, the disease ontologies identified in our screen had the highest alteration prevalence rates for *RAD51B* (uterus leiomyosarcoma) and *POT1* (angiosarcoma) among all sarcoma diseases surveyed, both within our cohort, as well as in independent cohorts^73,74^. In addition, we saw evidence that these alterations impacted telomeric content values when we analyzed the data pan-sarcoma. This suggests that the associations seen with telomere elongation within particular disease ontologies were observed due to the presence of sufficient sample counts and that the impact of these mutations may be extended to more diseases provided more samples are profiled, especially since the telomeric content values trended in the same direction in other sarcoma types (data not shown).

Overall, we found that alterations within *RAD51B*, *POT1*, *ATRX*, and *DAXX* were predominantly mutually exclusive, which frequently points to functional redundancy^75,76^, providing an additional layer of evidence that all four genes promote telomere elongation. On the other hand, *GID4* was co-altered with *ATRX* in 23% of *GID4* altered samples. However the presence of a *GID4* alteration on top of an *ATRX* alteration led to even higher telomeric content, within both soft tissue sarcoma nos and uterus leiomyosarcoma samples. This suggests either that GID4 activates an additional telomere elongation pathway or promotes the already-activated ALT pathway in those tumors.

Detecting telomere maintenance mechanisms may be beneficial in the prognosis of sarcomas. Specifically in osteosarcomas, the complete absence of a known telomere maintenance mechanism was strongly associated with improved survival^77^. In some reports, identifying the mechanism driving telomere elongation was linked to overall survival. For example, in soft tissue sarcomas, ALT was associated with decreased survival compared to patients with telomerase activation^26,78–82^, whereas in osteosarcomas, no difference in clinical outcomes was observed between ALT and telomerase-activated tumors^77^. In our analysis, we identified no difference in median overall survival from the date of metastatic diagnosis in patients with *GID4* altered sarcoma tumors compared to their WT counterparts.

It is important to note the limitations of this study. The FoundationOne®Heme testing platform only captures certain regions of the genome, thus our analysis was limited to those baited regions. Specifically, testing on this platform does not include baiting for either *TERC* or *TERT*, hence telomerase-mediated lengthening could not be assessed across these samples. However, analyzing samples from the independent validation datasets, sequenced on different panels, revealed that none of the samples altered in *RAD51B*, *GID4*, *ATRX*, or *DAXX* harbored alterations in either *TERC* or *TERT*. Furthermore, our results demonstrate a strong association between the observed alterations and high telomeric content. Future wet-lab studies are needed to confirm causation and elucidate the telomere maintenance mechanisms activated in these altered samples.

## Methods

### Foundation Medicine dataset

The Foundation Medicine sarcoma dataset consisted of 13555 sarcoma samples sequenced as a part of routine clinical care on the FoundationOne®Heme platform. All samples contained a minimum of 20% tumor nuclei. The pathologic diagnosis of each sarcoma case was first made by the referring institution and later confirmed by pathologists in Foundation Medicine on routine H&E staining. Samples represented 38 unique disease ontologies, with counts shown in Table S1. Counts are shown for sarcoma types with at least 5 samples. The top five disease ontologies present were soft tissue sarcoma (not otherwise specified, nos), soft tissue leiomyosarcoma, soft tissue liposarcoma, uterus leiomyosarcoma and bone osteosarcoma, accounting for 19.1%, 13.3%, 9.6%, 8.0% and 5.7% of the total sarcoma sample cohort, respectively. This is the main cohort referenced throughout the manuscript, unless otherwise indicated.

Approval for this study, including a waiver of informed consent and a HIPAA waiver of authorization, was obtained from the Western Institutional Review Board (Protocol No. 20152817) prior to study conduct. The Institutional Review Board granted a waiver of informed consent under 45 CFR § 46.116 based on review and determination that this research meets the following requirements: (i) the research involves no more than minimal risk to the subjects; (ii) the research could not practicably be carried out without the requested waiver; (iii) the waiver will not adversely affect the rights and welfare of the subjects. The work conforms to the principles of the Helsinki Declaration.

### Sample sequencing

The main cohort of samples were sequenced using a targeted panel (FoundationOne®Heme)^83,84^. Samples were assayed using baits for all coding exons of 465 cancer‐related genes plus select introns from 31 genes frequently rearranged in cancer. Sequencing of captured libraries was performed using the Illumina sequencing platform to a median exon coverage depth for targeted regions of ≥250X, and subsequently analyzed for genomic alterations, including short variant alterations (base substitution and indels), copy number alterations (focal amplifications and homozygous deletions), and select gene fusions or rearrangements. *TERC* and *TERT* were not included in the FoundationOne®Heme panel. Tumor mutational burden (TMB) was called as previously described^84^. Throughout this manuscript, “altered” refers to a sarcoma sample harboring known or likely pathogenic alterations in the indicated gene, whereas “WT” refers to a sample lacking these alterations or containing variants of unknown significance in the indicated gene.

### Telomeric content

We used TelomereHunter 1.1.0, a publicly available tool, for the estimation of telomeric content from targeted sequencing data^85^. TelomereHunter extracts telomeric reads and determines the telomeric content by normalizing the telomere read count by all reads in the sample with a comparable GC content (48–52%). We ran the tool on our sarcoma cohort using the default parameters and set the repeat threshold to 7 for 49 bp paired-end reads. For a subset of samples, RNA sequencing was also performed and TelomereHunter 1.1.0 was used for estimation of telomeric repeat-containing RNA (TERRA) content.

### Independent sarcoma datasets

For validation purposes, we utilized two distinct datasets. The first validation dataset consists of 1739 sarcoma samples sequenced on another panel, namely the FoundationOne®CDx (F1CDx) platform^83,84^ and is referred to as the F1CDx cohort. This panel utilizes a baitset targeting all coding exons of 309 cancer-related genes plus select introns from 34 genes frequently rearranged in cancer. Importantly, this panel includes baits for *RAD51B* and *GID4*, but not *POT1*. The second validation dataset, referred to as the Australian cohort, consists of 174 sarcoma samples profiled on the TSO500 panel, a targeted panel that contains baits for *RAD51B* and *GID4*, but not *POT1*^86^. Samples selected passed filters set for tumor purity and sequencing depth.

### Survival analysis of CGDB cohort

The retrospective clinical analysis utilized the nationwide (US-based) Foundation Medicine–Flatiron Health real-world clinico-genomic database (CGDB, data collected through December 31, 2021) which includes electronic health record (EHR)–derived deidentified data for patients in the Flatiron Health database who underwent comprehensive genomic profiling by Foundation Medicine, linked by de-identified deterministic matching^87^. Institutional Review Board approval of the study protocol was obtained prior to study conduct and included a waiver of informed consent based on the observational, non-interventional nature of the study (WCG IRB, Protocol No. 420180044).

Clinical outcome data were available for 449 saroma patients, 213 of which were soft tissue sarcoma patients. Overall survival was calculated from date of metastatic diagnosis based on a composite mortality variable^88^. To account for left truncation, patients were treated as at risk of death only after the later of their first sequencing report date and their second visit in the Flatiron Health network on or after January 1, 2011, as both are requirements for inclusion in the cohort. For the Kaplan–Meier analyses, the log-rank test was used to compare between *GID4* WT vs altered. Due to low sample count, survival analysis couldn’t be performed for *RAD51B* or *POT1*.

### Survival analysis of Australian cohort

Clinical outcome data were available for 235 sarcoma patients, 211 of which were soft tissue sarcoma patients. Overall survival was calculated from date of metastatic diagnosis. For the Kaplan–Meier analyses, the log-rank test was used to compare between *GID4* WT vs altered. Due to low sample count, survival analysis couldn’t be performed for *RAD51B* or *POT1*.

### Statistics

All statistical analyses were conducted using R (4.0.2)^89^. Wilcoxon rank sum (two-sided) and Kruskal-Wallis were used to test for differences between two groups or between multiple groups, respectively. To screen for genetic alterations associated with high telomeric content, we first identified samples with telomeric content values in the top quartile of their respective disease and those with low content in the bottom quartile and then performed a Fisher’s exact test to check for the enrichment of alterations across the baited genes. Results for all gene hits with a corrected *P* value < 0.05 after Bonferroni’s correction and an odds ratio >1 are plotted in Fig. 1B. For the analysis shown in Fig. 2F, we performed a Fisher’s exact test to check for the enrichment of amplifications across chromosome bands of chr17p in samples with telomeric content in the top quartile. MEGSA (version beta 2) was used to test for mutual exclusivity in Fig. 3B^90^. To denote significance, *p < 0.05, **p < 0.01, ***p < 0.001, and ****p < 0.0001, while ns denotes non-significant.

### Reporting summary

Further information on research design is available in the Nature Research Reporting Summary linked to this article.

### Supplementary information

Supplementary figures

Reporting Summary

Supplementary table 1

Supplementary table 2

Supplementary table 3

Supplementary table 4

## Data Availability

Academic researchers can gain access to Foundation Medicine data in this study by contacting the corresponding author and filling out a study review committee form. You and your institution will be required to sign a data transfer agreement. Survival data that support the findings of this study have been originated by Flatiron Health, Inc. and Foundation Medicine, Inc. These de-identified data may be made available upon request and are subject to a license agreement with Flatiron Health and Foundation Medicine; interested researchers should contact cgdb-fmi@flatiron.com to determine licensing terms.
