# Supplementary material for: A pan-sarcoma landscape of telomeric content shows that alterations in RAD51B and GID4 are associated with higher telomeric content
Source: NPJ Genom Med. 2023 Sep 14;8:26. doi: 10.1038/s41525-023-00369-6 (PMC10502097; doi:10.1038/s41525-023-00369-6)
Supplement: Supplementary file 1 — Supplementary figures [file 41525_2023_369_MOESM1_ESM.pdf]

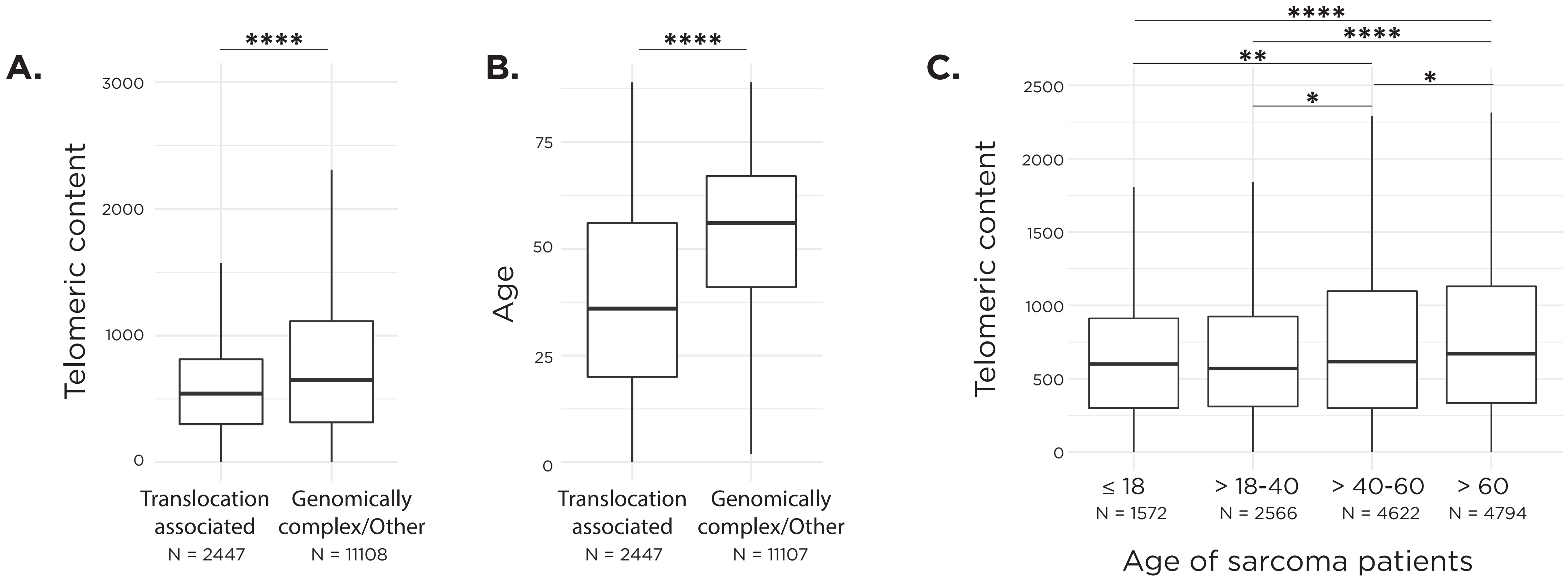

Supplementary Figure 1

Boxplot displaying the distribution of telomeric content values (A) and age (B) of samples within translocation-associated diseases vs genomically complex/other sarcoma diseases. C. Boxplot showing the telomeric content of samples by age group. * denotes $p<0.05$, ** denotes $p<0.01$, and **** denotes $p<0.0001$.

DAXX

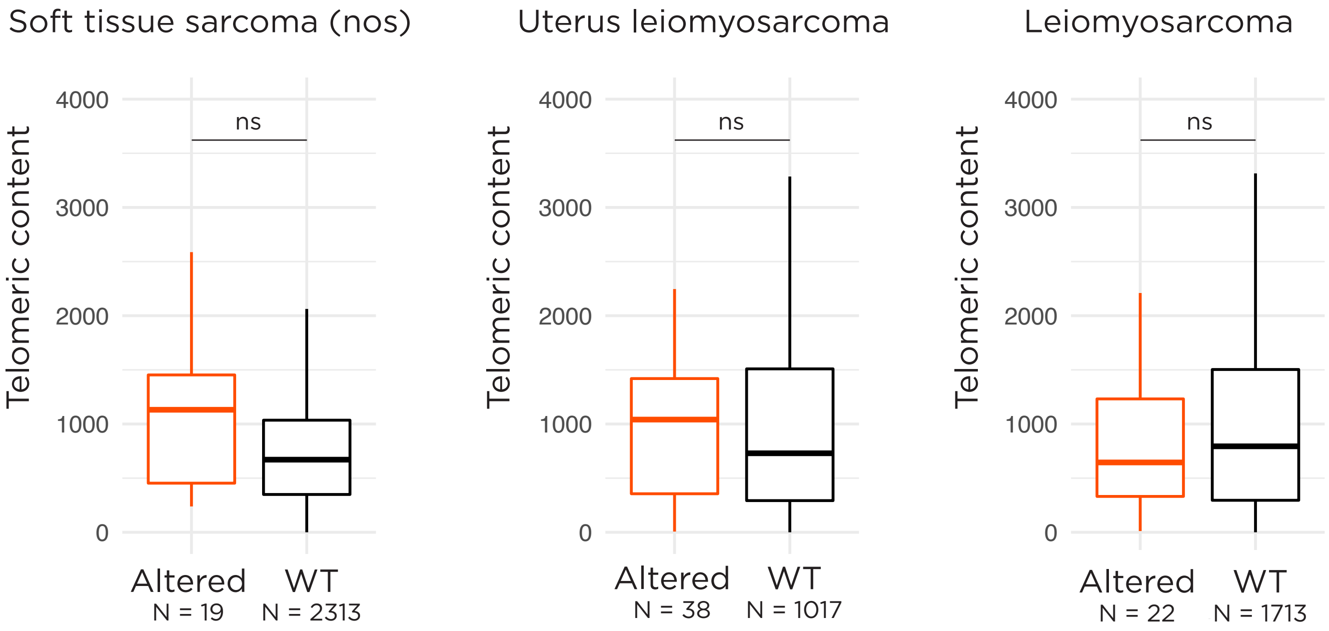

Supplementary Figure 2
The impact of alterations in *DAXX* on the telomeric content values of samples within diseases harboring at least 20 altered samples, yet not identified in our screen. ns, not significant.

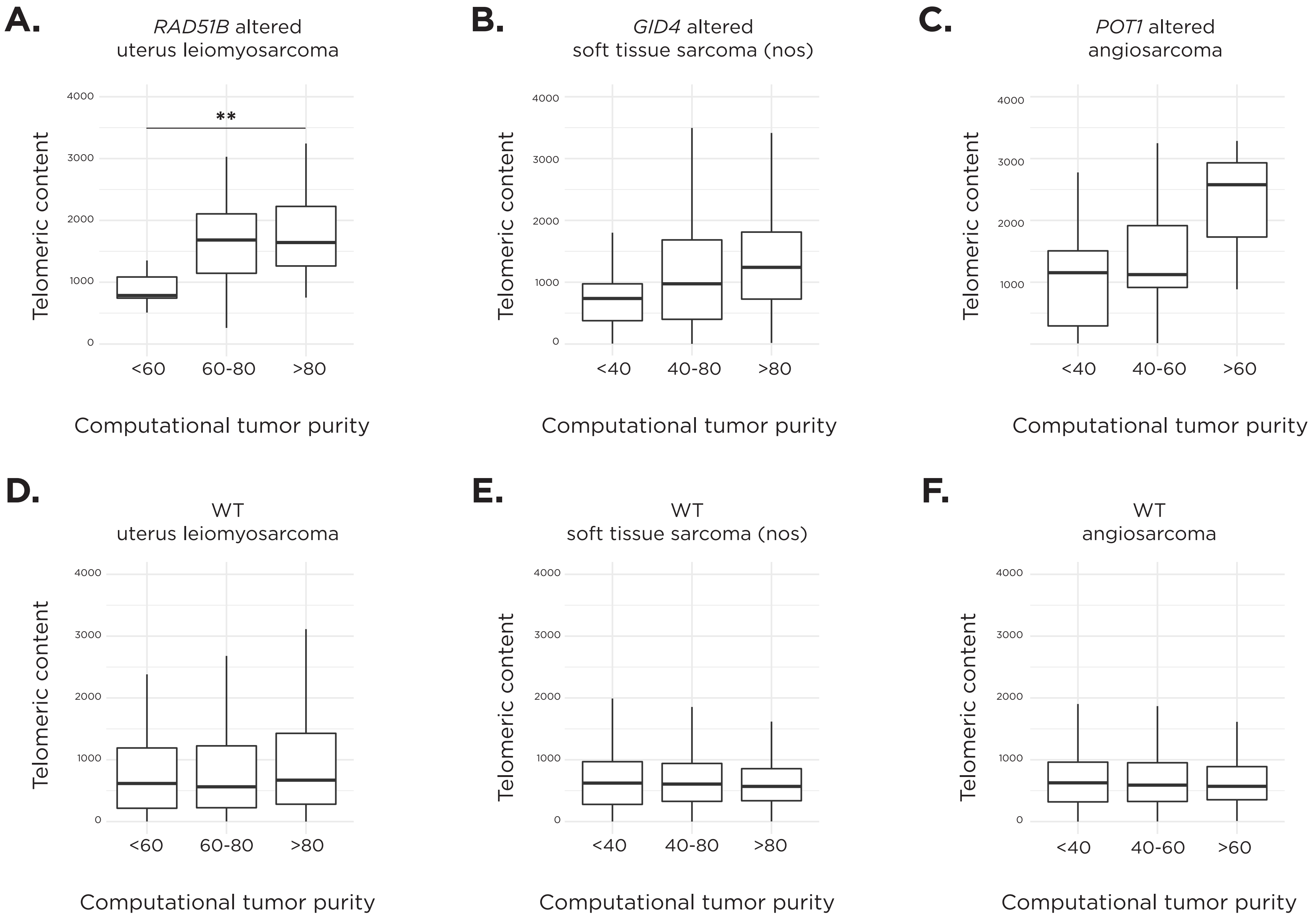

Supplementary Figure 3
Boxplots displaying the distribution of telomeric content values across samples within each computational tumor purity bin for *RAD51B* altered uterus leiomyosarcoma (A), *GID4* altered soft tissue sarcoma nos (B), and *POT1* altered angiosarcoma (C), as well as WT uterus leiomyosarcoma (D), soft tissue sarcoma nos (E), and angiosarcoma (F). WT indicates that samples lack known/likely alterations in any of the telomere-maintenance mechanism genes described in this work.

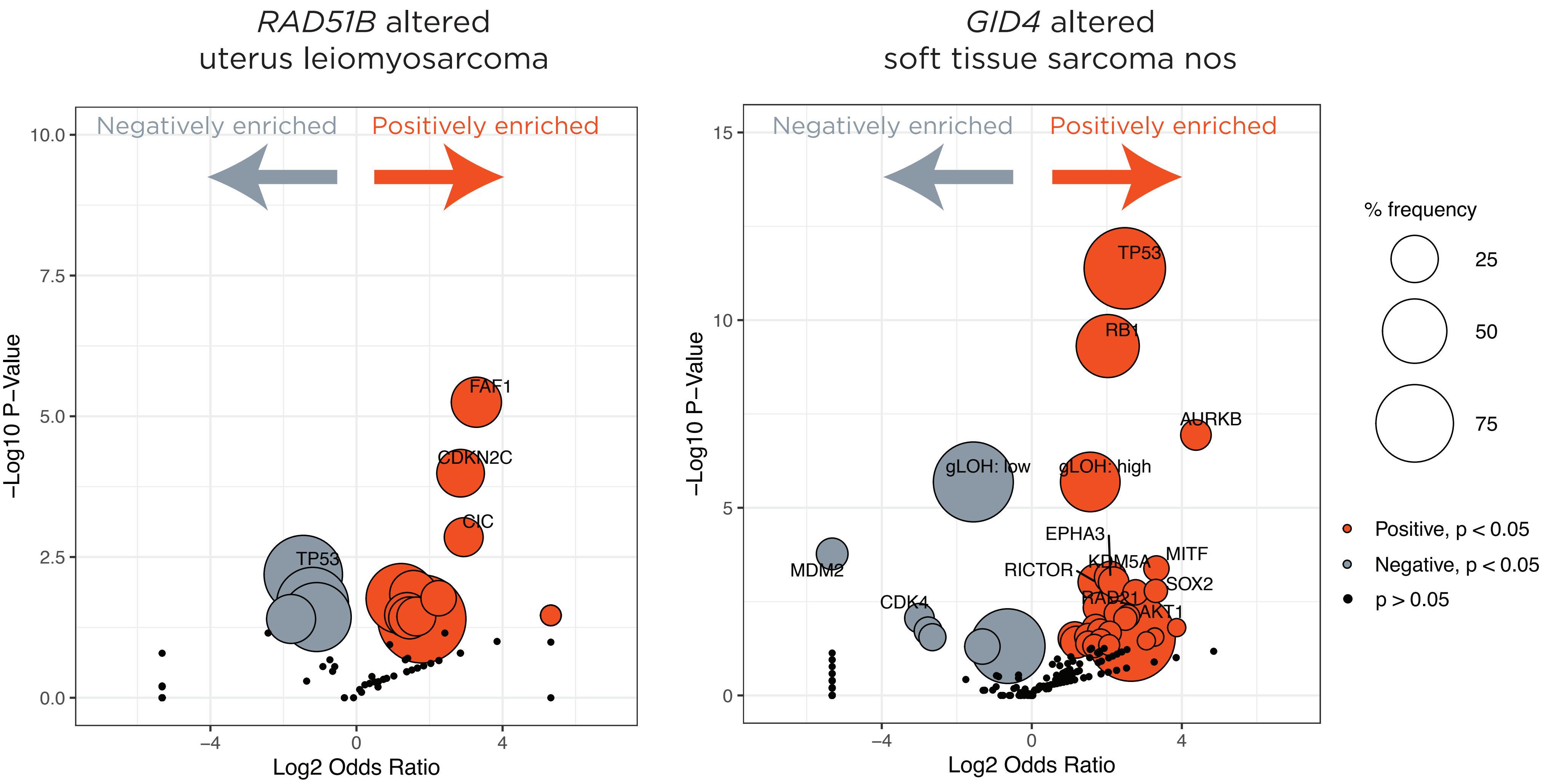

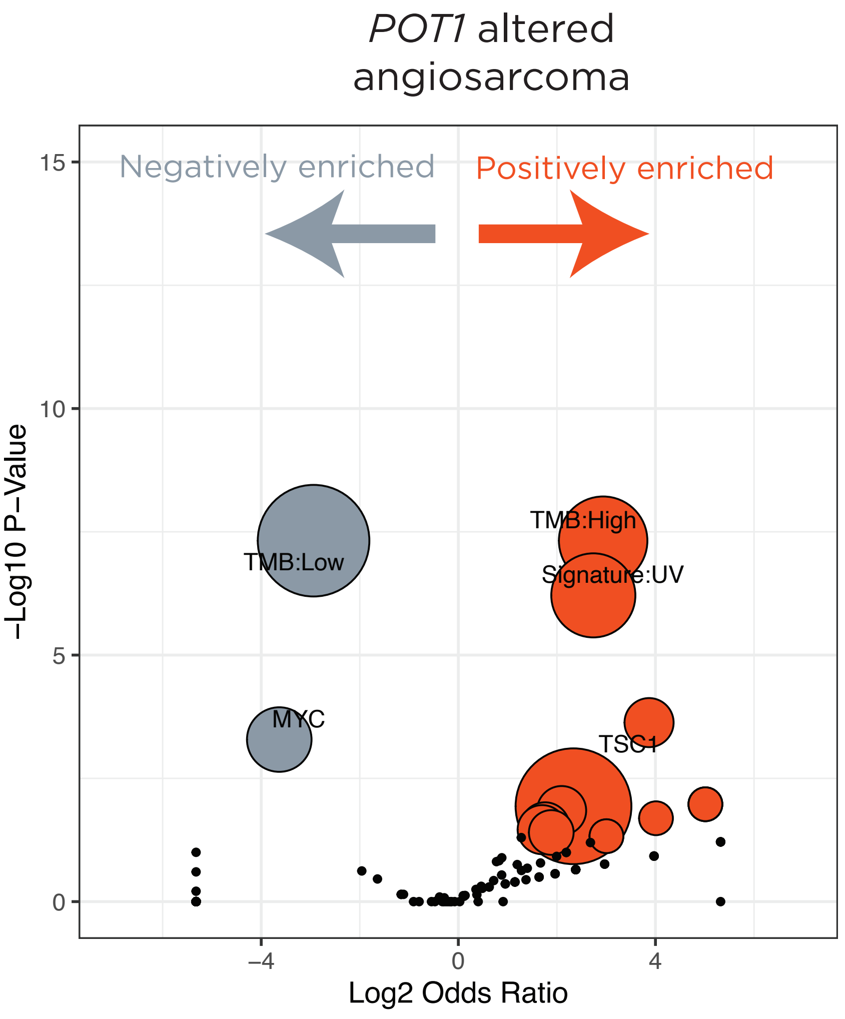

Supplementary Figure 4
Volcano plots showing the enrichment of genetic alterations and features across *RAD51B* altered uterus leiomyosarcoma, *GID4* altered soft tissue sarcoma nos, and *POT1* altered angiosarcoma. TMB, tumor mutational burden.

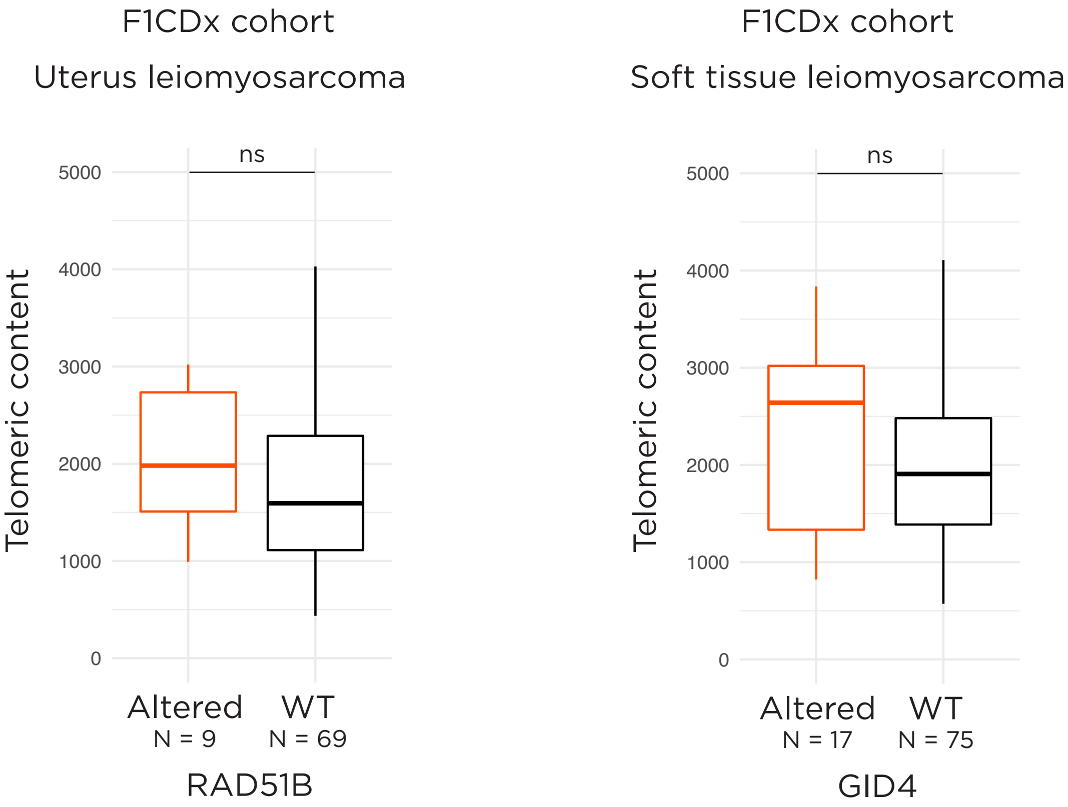

Supplementary Figure 5
Boxplots displaying the telomeric content of samples profiled on the FoundationOne®CDx assay (F1CDx), specifically *RAD51B* altered vs *RAD51B* WT uterus leiomyosarcoma samples and *GID4* altered vs *GID4* WT soft tissue leiomyosarcoma samples. ns, not significant.

All sarcomas

CGDB cohort

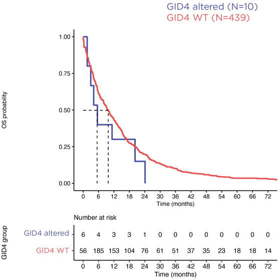

Australian cohort

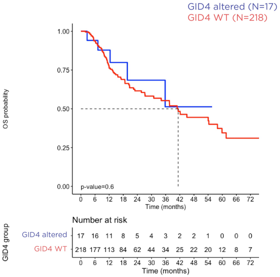

Soft tissue sarcomas

CGDB cohort

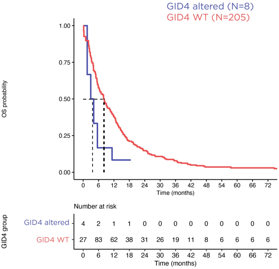

Australian cohort

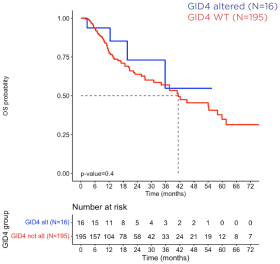

Supplementary Figure 6
Kaplan-Meier survival curves for patients with GID4 WT vs altered sarcoma tumors in the CGDB cochort and the Australian cohort. Survival curves are also shown specifically for patients with soft tissue sarcomas.
